# Metacontrol Regulates Creative Thinking: An EEG Complexity Analysis Based on Multiscale Entropy

**DOI:** 10.3390/brainsci14111094

**Published:** 2024-10-30

**Authors:** Hang Qi, Chunlei Liu

**Affiliations:** School of Psychology, Qufu Normal University, Qufu 273165, China; qihang@qfnu.edu.cn

**Keywords:** metacontrol, creativity, electroencephalography, multiscale entropy, neural networks

## Abstract

Previous studies have shown that creative thinking is associated with metacontrol, but its neural basis is unknown. The present study explored the neural basis of both by assessing EEG complexity through multiscale entropy. Subjects were engaged in a metacontrol task and an Alternative Uses Task, grouped according to task performance, and the EEG was analysed by multiscale entropy. The results showed that EEG complexity was significantly higher in the high-metacontrol and high-creativity groups than in the low-metacontrol and low-creativity groups, respectively, at high time scales. The metacontrol adaptability score and multipurpose task score were significantly and positively correlated with the EEG complexity at multiple electrode sites. It suggests that metacontrol and creativity are dependent on the activation of long-duration neural networks.

## 1. Introduction

Creative thinking, as a higher cognitive function, is key to exploring uncharted territory, driving innovation and generating unique insights. It not only promotes understanding of the nature of things but also stimulates new ideas with social value. With advances in the field of neuroscience, especially the application of neuroimaging techniques such as EEG and fMRI, researchers have been able to delve deeper into the brain mechanisms behind creative thinking [1]. These studies have revealed the relationship between activation in different regions of the brain and creative thinking and the neural networks involved. However, most of the existing studies have focused on the activation of specific brain regions, ignoring the complexity of creative thinking as a whole dynamic process. In addition, most studies have used traditional time or frequency domain analyses, which may fail to capture the complexity and dynamics of creative thinking across time scales.

In order to perform purposeful behaviours, the human brain needs to adjust its information processing according to its goals, and these adjustments are collectively known as cognitive control [2]. Metacontrol is the process of monitoring and adjusting cognitive control. Examples of metacontrol include flexibility and stability in allocating attention between established and new goals; exploitation versus exploration in trade-offs between known actions and exploring new options; and immediate versus delayed gratification in trade-offs between long-term goals and immediate gratification, among others [3,4]. Creative activity encompasses a multifaceted array of cognitive processes, including the collection, analysis, and dissemination of information [5]. For instance, in order to generate an original concept or product, an individual must possess both flexible cognitive control to think in an unconventional manner and to establish novel connections between concepts and stable cognitive control to focus on the information at hand and to persevere in working through it in order to make decisions and ultimately to create a comprehensive solution. Metacontrol is of pivotal importance in the coordination and balancing of these two opposing types of cognitive control [6]. Hommel’s metacontrol state model further elucidates the competitiveness of solution choices in creative activity and that this competitiveness is moderated by metacontrol [7]. While this idea is theoretically supported, the neural mechanisms behind it remain unclear.

There are fewer existing studies on the neural mechanisms of metacontrol. Kirschner and colleagues found that misperception affects the balance between active and passive control and that misperception is associated with Pe and ERN in the event-related locus coeruleus (ERP) [8]. These studies have focused on localised brain regions and single time scales, whereas metacontrol processes actually involve collaboration between different regions of the brain and different time scales [9]. Similarly, creative thinking has been shown to involve the synergistic action of multiple regions of the brain, which involves not only the activity of specific functional areas but also neural communication across regions [10,11,12]. EEG signals provide a window into this nonlinear, dynamically changing process [13,14]. The neural complexity (Cn) measure proposed by Tononi analyses neural signals through information entropy, revealing a balance between local functional separation and overall functional integration [13]. Several studies have explored the physiological significance of neural signalling complexity embodied in functional coupling between different brain regions, adaptation to external stimuli, the effect of noise on information processing within the system and the modulation of interregional synchrony [15,16,17,18,19]. However, traditional complexity measures are sensitive to noise and require large amounts of noise-free data. In contrast, the improved sample entropy (SampEn) is robust to noise and suitable for EEG data analysis [20].

In neural networks, interactions between different functional regions involve different distances and types of connections. This predicts that dynamic processes of thinking activities on multiple time scales may involve different functions [21]. In practice, these dynamic processes are manifested in the simultaneous activity of multiple frequency bands, with different frequency bands reflecting different ranges of activation in neural networks [22,23]. Given that metacontrol and creative thinking involve connections both within and across neural subsystems [11,12,24], the measurement of their EEG complexity should be performed on multiple time scales. Multiscale entropy was introduced by Costa. The MSE method, based on an improvement of sample entropy, aims to understand the complexity of biological signals on different time scales [25]. MSE has been applied to study the brain dynamics of psychiatric disorders such as schizophrenia and depression [26] but has not yet been widely used in the study of the neural mechanisms of creative thinking. Therefore, MSE provides a new perspective in analysing the neural mechanisms of metacontrol regulation of creative thinking.

In summary, multiscale entropy analysis, which can observe brain activity in terms of whole-brain connectivity and multiple time scales, may be important for understanding complex cognitive activities such as metacontrol and creative thinking that involve multiple brain regions. The research hypothesis is that there are specific neurodynamic features of metacontrol and creative thinking processes that can be captured by multiscale entropy analysis methods.

## 2. Methods

### 2.1. Subjects

Applying G*Power software 3.1, 52 subjects (26 in each group) were needed for the independent samples *t*-test at a statistical test power (statistical power) of 0.8, an alpha level of 0.05, and an effect size of 0.4 (medium effect size) [27]. Sixty-four current university students were recruited. Subjects were paid in cash for participating in the study. A total of six subjects were excluded from further analyses, four due to discontinuing midway and not completing all tasks, and two due to not understanding the requirements of the task. Thus, the final analysis sample consisted of 58 subjects (41 women, M_age_ = 20.63, SD_age_ = 2.02). All subjects were right-handed, mentally healthy, had normal vision or corrected vision, and had no experience participating in similar experiments.

### 2.2. Experimental Materials

#### 2.2.1. Metacontrol Task

**Figure 1:**
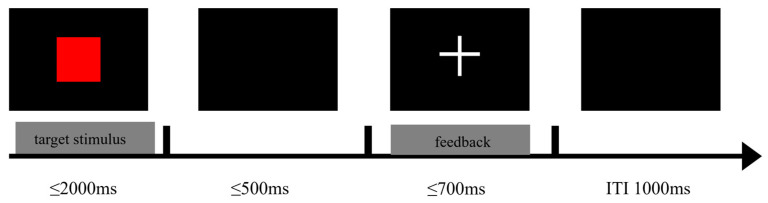
Metacontrol task process.

An adapted version of the experimental paradigm developed by Bocanegra and Hommel was used in this study [28]. The paradigm consists of two tasks, the ‘easy’ task and the ‘difficult’ task. The flow of the experiment is shown in Figure 1. A 500 ms gaze point (‘+’) was first presented in the centre of the screen, followed by a 2000 ms visual stimulus. The stimuli varied in shape (square or diamond), colour (green or red), and size (small: 2.5 cm diameter or large: 5 cm diameter). All possible combinations of stimulus features were randomised and appeared with equal frequency in both tasks. In the simple task, subjects were asked to respond to the shape of the target stimulus by pressing the ‘f’ key when the target stimulus was a square and the ‘j’ key when the target stimulus was a diamond. In the difficult task, subjects were asked to respond to a characteristic combination of size and colour of the target stimulus. The ‘f’ key was pressed when the target stimulus was large and red or small and green, and the ‘j’ key was pressed when the target stimulus was large and green or small and red. Trials that did not respond after a certain period of time were regarded as incorrect responses. After the stimulus response, a blank screen was presented for 700 ms, and after the blank screen, feedback was presented for 500 ms, with ‘+’ (positive feedback) when the subject pressed the key correctly and ‘−’ (negative feedback) when the subject pressed the key incorrectly. A blank screen was presented for another 1000 ms, after which the next trial was started. Subjects were asked to perform a practice experiment before each task and did proceed to the formal experiment until the practice experiment was at least 80% correct.

A fixed sequential order design was used as the study was concerned with the ability to generate adaptive control in response to different control demands, rather than the ability to flexibly switch between different control demands [29]. In order to keep potential order effects constant, all subjects were asked to complete the difficult task before the easy task.

The ratio of subjects‘ performance in completing the task was the correctness of subjects’ responses to the stimulus (ACC) divided by the reaction time (RT), with larger results indicating (relatively) better performance. Subjects’ adaptive scores were calculated by subtracting their performance ratios on the easy and difficult tasks, and this score can account for the relationship between reaction time and correctness, as well as the difference in performance between the two tasks. Therefore, this study categorised subjects into high- and low-metacontrol groups by the median adaptive score. Subjects who performed better on the simple task and showed large task differences were categorised into a high-metacontrol group (high-adaptability group), and subjects with smaller task differences were categorised into a low-metacontrol group (low-adaptability group). Previous research has found that compared to low-metacontrol individuals, high-metacontrol individuals are able to flexibly adapt to different cognitive demands, effectively reduce top-down control when cognitive control demands are low, and perform better in tasks, especially in simple tasks where the task performance difference between the two is greater [30].

#### 2.2.2. The Alternative Uses Task (AUT)

**Figure 2:**
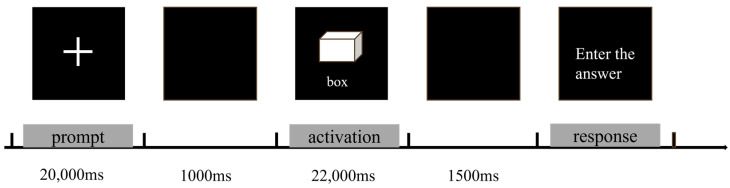
AUT task process.

The Alternative Uses Task (AUT) assesses the subjects’ ability to generate novel and practical uses for an item within a limited time frame. In order to increase the process of refinement and evaluation of the subjects’ creative thinking process, the subjects were asked in the instructions to think of as many creative uses as possible in the allotted time and finally choose to input one of the most original ideas [31,32]. The specific flow of the experiment was as follows: first, the gaze point ‘+’ was presented in the screen for 20,000 ms (prompt phase 1000–21,000 ms), followed by an empty screen for 1000 ms, and then a picture of an everyday object and its name (e.g., a cardboard box) was presented, and subjects were asked to think about as many creative uses for the object as possible The presentation time was 22,000 ms (Figure 2). When the time was up, the picture disappeared and an answer prompt was presented, in which subjects entered the most creative use they could think of and then pressed the ‘Enter’ key to move on to the next question, with an interval of 2000 ms. Before entering the formal experiment, subjects were required to complete a practice experiment to familiarise themselves with the rules and procedures of the task. In order to assess the originality of the subjects’ answers, we used the consensus assessment technique (CAT), in which four trained evaluators scored the originality of each answer on a five-point Likert scale, with a score of 1 indicating “not at all original”, 2 indicating “not very original”, 3 indicating “generally original”, 4 indicating “fairly original”, and 5 indicating “highly original”. The originality score is the average of the scores of the subjects’ ideas on each topic.

### 2.3. Recording and Analysis of EEG Data

EEG signals were acquired using the Brain Products system and the International 10–20 System Extended 64 Conductive Polar Cap. Brain Products’ ActiCHamp active channel amplifier was used to amplify the signals. Sampling rates of 1000 Hz on-line recording, 0.1–100 Hz on-line filtering, and 50 Hz trap filtering were used. Point FCz was used as the online reference electrode and point FPz as the ground electrode, and all electrodes had a head scalp resistance impedance of less than 5 KΩ. Data were analysed offline using BrainVision Analyzer 2.1 software. Firstly, binaural mastoid averaging was used as a heavy reference. Band-pass filtering from 0.1 to 40 Hz and trap filtering at 50 Hz were performed. Then Independent Component Analysis (ICA) was used to exclude artefacts. For the metacontrol task, the time window from 500 ms before stimulus presentation to 2000 ms after stimulus presentation (−500–2000 ms) was selected for segmentation, with 500 ms before the stimulus presentation designated as the baseline correction. For the AUT task, a time window from 500 ms before stimulus presentation to 20,000 ms after stimulus presentation (−500–20,000 ms) was selected for segmentation, with 500 ms before stimulus presentation as the baseline correction.

### 2.4. Multiscale Entropy Calculation

Multiscale entropy is a measure that describes the complexity of a time series from multiple time scales on the basis of sample entropy. Firstly, for a one-dimensional time series {*x*_1_,…, *x_i_*,…, *x*_N_}, a coarse-graining process is performed to divide it into non-overlapping time windows, each of which is of length *τ*. Second, the data points within each window are averaged to obtain the coarse-grained time series. Each element in the coarse-grained time series can be calculated by the following formula:

$$y_{j}^{\left(\tau \right)}=\sum_{i=(j-1)\tau +1}^{j\tau }x_{i}\;1\;\leq \;j\;\leq \;N/\tau$$

The length of the coarsely granulated time series is equal to the length of the original series divided by the window length, and when *τ* = 1, the coarsely granulated time series is equal to the original time series. Second, each coarsely granulated time series is measured using sample entropy (SampEn) [20]. SampEn measures the probability that two sequences of m data points that are similar to each other (within a given tolerance r) will remain similar at the next point (m + 1) in the data set (N), where N is the time series length. A higher SampEn indicates a lower regularity of the time series, i.e., the same pattern is rarely repeated in that time series. The SampEn under each window length is aggregated to obtain the final multiscale entropy. If the entropy of one time series is higher than another time scale under most window lengths, the former is considered more complex than the latter. The choice of time scale is usually clustered at 15–40, and to facilitate comparison with previous studies, the time scale in this study was set at 20 [33,34,35,36].

The processing of the EEG data and the calculation of the multiscale entropy were performed in matlab 2019a, and the code for the multiscale entropy calculation was provided by Costa [29].

### 2.5. Power Analysis

Spectral power analysis, as a traditional EEG analysis method, is analyzed here as a comparison with multiscale entropy analysis. The spectral density was calculated by Fast Fourier Transform in matlab, and the absolute power was calculated for the consecutive 10 s of data since the stimulus appeared, and then the spectrum was divided into five bands of δ (2–4 Hz), θ (4–8 Hz), α (8–13 Hz), β (13–30 Hz), and γ_low_ (30–40 Hz), and the relative power of each band was its absolute power divided by the total power.

### 2.6. Functional Network Analysis

Multiscale entropy analysis was performed for each brain functional network using the algorithm of Giacometti to assign electrodes to each network according to a probability map [36,37].

### 2.7. Statistical Analysis

Statistical analyses were performed using the R 4.3 adaptive scores and SampEn values for each SF were normally distributed (tested using the Shapiro–Wilk test). For MSE analyses, a repeated-measures ANOVA with group as a between-subjects factor and SF and electrode point as within-subjects factors was performed to test for differences. For relative efficacy analyses, we performed independent samples *t* tests. Greenhouse–Geisser correction was applied to ANOVAs that did not meet the assumption of sphericity. The Bonferroni method was used for post hoc comparisons. The significance of Pearson correlation coefficient is corrected using the Benjamini–Hochberg method. For ANOVA, a two-tailed level of 0.05 was considered statistically significant, and for post hoc *t*-tests, a two-tailed level of 0.01 was considered statistically significant.

## 3. Experimental Results

### 3.1. Behavioural Data

Subjects were categorised into high- and low-metacontrol groups based on the median adaptive scores of the metacontrol task, with 29 in the high-metacontrol group and 29 in the low-metacontrol group. In order to test the validity of the grouping, two-factor repeated measures analyses of variance (ANOVAs) of group (high-metacontrol group, low-metacontrol group) × task (difficult task, easy task) were performed on the subjects’ performance ratios (i.e., ACC/RT) on the metacontrol task.

Adaptive score = ACC_simple_/RT_simple_ − ACC_difficult_/RT_difficult_

**Figure 3:**
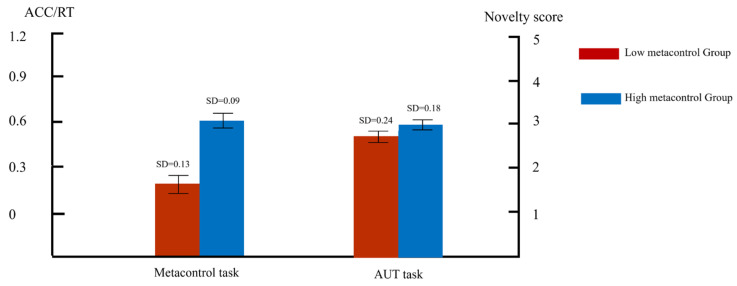
Performance ratio of high- and low-metacontrol groups in metacontrol tasks (ACC/RT) and novelty score in AUT tasks.

Results indicated a significant main effect of task (*F*(1, 58) = 117.14, *p* < 0.001, η_p_^2^ = 0.52), with subjects performing significantly better on the easy task (M = 0.176, SD = 0.03) than the difficult task (M = 0.138, SD = 0.02). The main effect of metacontrol group was significant (*F*(1, 58) = 40, *p* < 0.001, η_p_^2^ = 0.28), with subjects in the high-metacontrol group performing significantly better than those in the low-metacontrol group. The interaction between the task groups was significant, (*F*(1, 58) = 28.65, *p* < 0.001, η_p_^2^ = 0.19). Further simple effects analyses indicated that the high-metacontrol group (M = 0.199, SD = 0.02) significantly outperformed the low-metacontrol group (M = 0.153, SD = 0.02) in the simple task (*p* < 0.001); in the difficult task, the difference between the high- and low-metacontrol groups was not significant (*p* = 0.074). Overall, the analysis of the behavioural data suggests that the median adaptive scores through the metacontrol task can effectively classify subjects into high- and low-metacontrol groups (Figure 3).

In the AUT task, a synoptic assessment technique was adopted and four trained raters were invited to rate the novelty of all answers from all subjects on a five-point scale. With a compliant internal consistency coefficient (ICC(2,k) = 0.844), subjects’ final score in the AUT task was the mean of the ratings given by the four raters. An independent samples *t*-test on the novelty of the AUT for subjects in the high- and low-metacontrol groups showed that subjects in the high-metacontrol group had significantly higher novelty scores (M = 2.98, SD = 0.18) than the originality scores of subjects in the low-metacontrol group (M = 2.74, SD = 0.24), *t*(58) = 2.00, *p* < 0.001, Cohen’s d = 1.14). The results suggest that subjects in the high-metacontrol group outperformed subjects in the low-metacontrol group on the AUT task.

### 3.2. Electroencephalographic Data

#### 3.2.1. Metacontrol Task

**Figure 4:**
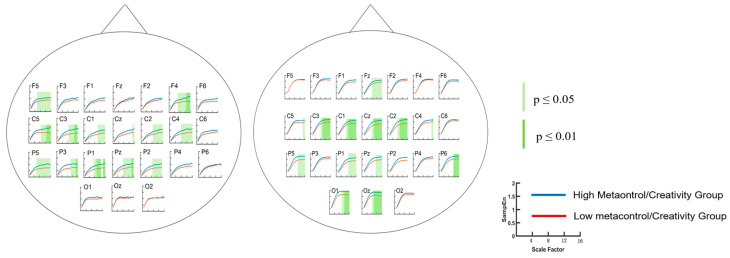
Multiscale entropy of EEG signals in the metacontrol task (**left**) and AUT task (**right**). Time scales with significant differences were marked with different depths of green respectively.

**Figure 5:**
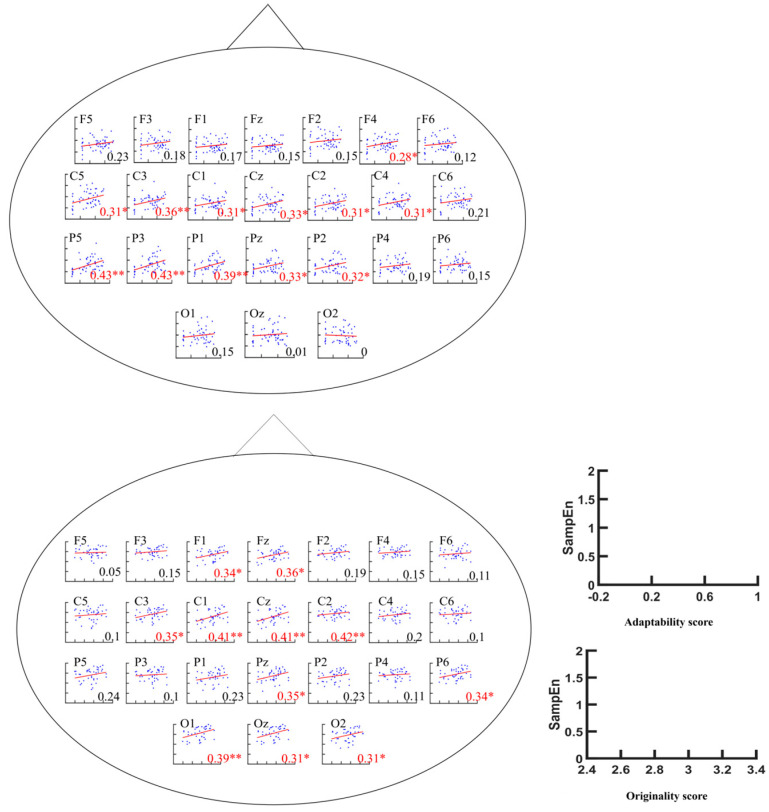
Pearson correlation between the adaptability score (**top**) and originality score (**bottom**) of the metacontrol task and multiscale entropy. (*) means *p* < 0.05, (**) means *p* < 0.01.

**Figure 6:**
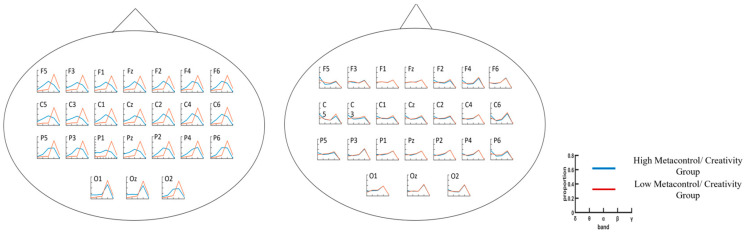
Relative power analysis of metacontrol task (**left**) and AUT task (**right**). Each panel presents the average of relative power across delta, theta, alpha, beta and gamma(low) bands.

A three-way analysis of variance (ANOVA) was conducted with sample entropy (SampEn) as the dependent variable and metacontrol group, electrode point, and time scale (SF) as the independent variables. The ANOVA results showed a significant main effect of electrode point (*F*(23, 19,200) = 73.2, *p* < 0.001, η_p_^2^ = 0.08) and a significant main effect of group (*F*(1, 19,200) = 77.04, *p* < 0.001, η_p_^2^ = 0.004); the main effect of SF was significant (*F*(15, 19,200) = 767.91, *p* < 0.001, η_p_^2^ = 0.375), the interaction of group × electrode site × SF was not significant (*F*(345, 19,200) = 0.07), and the interaction of group × SF was not significant (*F*(15, 19,200) = 1.09, *p* = 0.358). The group × electrode site interaction was significant (*F*(23, 19,200) = 4.11, *p* < 0.001, η_p_^2^ = 0.005), and the electrode site × SF interaction was not significant (*F*(345, 19,200) = 0.56). Unpaired *t*-tests were performed for the high/low-metacontrol ability groups under each electrode point, and the results of the metacontrol task showed a significant increase in multiscale entropy in parts of the central, parietal, temporal, and frontal lobes for the high-metacontrol group at higher time scales (Figure 4). Correlation analyses showed that metacontrol scores were significantly and positively correlated with SampEn values at multiple electrode sites (F4, Cz, C1, C2, C3, C4, C5, Pz, P1, P2, P3, and P5) (Figure 5). Spectral analysis of the data from each electrode point revealed significant differences between the two groups of subjects in the frontal, parietal, and temporal lobe regions, with the low-metacontrol group having a higher relative power of the beta band (13–30 Hz) than the high-metacontrol group (Figure 6).

#### 3.2.2. AUT Task

Subjects were categorised into high-creativity group/low-creativity group based on the median originality score of all subjects. A three-way analysis of variance (ANOVA) was conducted with sample entropy (SampEn) as the dependent variable and creativity group, electrode point, and time scale (SF) as the independent variables, and according to the ANOVA analysis, there was a significant main effect of electrode point (*F*(23, 19,200) = 51.33, *p* < 0.001, η_p_^2^ = 0.058), a significant main effect of time scale (*F*(15, 19,200) = 1634.61, *p* < 0.001, η_p_^2^ = 0.56), a significant main effect of group (*F*(1, 19,200) = 1905.19, *p* < 0.001, η_p_^2^ = 0.09), a nonsignificant interaction of group × SF × electrode point (*F*(345, 19,200) = 0.05), a significant interaction of group × SF (*F*(15, 19,200) = 7.22, *p* < 0.001, η_p_^2^ = 0.006), a significant electrode point × SF interaction (*F*(345, 19,200) = 1.13, *p* = 0.049, η_p_^2^ = 0.02), and a significant electrode point × group interaction (*F*(23, 19,200) = 9.15, *p* < 0.001, η_p_^2^ = 0.01). In the AUT task, the high-creativity group had significantly higher multiscale entropy than the low-creativity group on medium and high time scales, and the regions with significant differences in multiscale entropy were distributed in the central, parietal, temporal, and occipital lobes (Figure 4). Correlation analyses showed that AUT task result originality scores were significantly and positively correlated with SampEn values at Fz, F1, Cz, C1, C2, C3, Pz, P6, Oz, O1, and O2 (Figure 5).

#### 3.2.3. Functional Network Analysis

**Figure 7:**
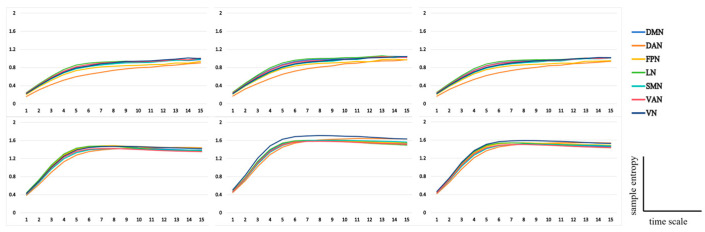
Multiscale entropy of the brain functional network in metacontrol task (**top**) and AUT task (**bottom**), From left to right are the high creativity/metacontrol group, low creativity/metacontrol group, and overall average.

Independent sample *t*-tests were performed on the different brain functional networks with group as the independent variable and the sum of multiscale entropy at 10–15 time scales as the dependent variable. The functional networks included in the statistics include default mode network (DMN), saliency network, dorsal attention network (DAN), frontoparietal network (FPN), ventral attention network (VAN), visual network (VN), somatic motor network (SMN), and limbic network (LN) (Figure 7).

## 4. Discussion

MSE, as a tool to reveal the performance of EEG complexity on multiple time scales, provides researchers with a new way to observe brain activity. The results of the study showed that the high-metacontrol group performed significantly better than the low-metacontrol group in the creativity task, and that the EEG complexity of the high/low-metacontrol group and the high/low-creativity group differed significantly at medium and high time scales. Levels of creativity and metacontrol were significantly correlated with EEG complexity at high time scales, i.e., higher levels of creativity and metacontrol were associated with higher complexity of corresponding EEG activity. There were significant differences in the EEG complexity of the dorsal attentional network (DAN) across groups in the metacontrol task and in the EEG complexity of all functional brain networks in the creativity task. The significant differences in EEG complexity occurred only at medium to high time scales (10–15), suggesting that long duration neural network activation may underlie the neural basis of metacontrol and creativity. And the increased EEG complexity of specific functional brain networks may suggest a role for these networks in different cognitive activities.

Previous studies have shown that creative activity involves the synergistic action of multiple neural networks. For example, Jung found that cortical thickness of a specific neural network was positively correlated with creativity scores and that the network was not limited to specific regions of the brain [38]. Studies by Levine and Kowatari also revealed that creativity is related to the collaboration of multiple regions of the brain [24,39]. the resting-state fMRI study further indicated that long-distance functional connectivity between the medial prefrontal and posterior cingulate cortex and middle temporal gyrus plays a key role in creative activity [40]. EEG studies have also found that neural network activation during creative tasks is not only confined within the hemispheres but also crosses hemispheres and occurs mainly at long distances between brain regions [11,12]. Hommel’s theory of metacognitive control emphasises the importance of flexible metacognitive state regulation for creativity but does not specify which regions of the brain are involved in these abilities [7]. Beaty’s study, on the other hand, proposes that creativity is influenced by the executive control network (ECN) in conjunction with the default mode network (DMN), and that these regions may influence creativity through their role in metacognition [41].

EEG complexity can provide richer neuroscience information at multiple time scales. Small clusters of neurons produce high-frequency oscillations, and large clusters of neurons produce low-frequency oscillations [23,42]. Thus, neural signal complexity on low time scales reflects small-scale neural network properties, and complexity on high time scales reflects large-scale neural network properties [21]. Differences in EEG complexity between groups were only significant at high time scales, possibly indicating that metacontrol and creativity-related brain activity are associated with remote neural network activation, consistent with previous findings.

Further functional brain network analyses revealed significant differences between groups in the EEG complexity of the dorsal attentional network (DAN), a persistent bilateral network responsible for attention maintenance and top-down control (see Table 1). Compared to the low-metacontrol group, the high-metacontrol group showed higher EEG complexity on medium to high time scales, suggesting a higher intensity of interactions at moderate distances within the network and between remote neuronal collections in other brain networks. This finding implies that top-down cognitive control may play an important role during flexible and persistent metacontrol state switching.

In the creativity task, there were significant differences between the high- and low-creativity groups in the EEG complexity of all functional brain networks, especially in the visual network (VN) (see Table 2). This implies that creative activity may involve neural activity in multiple regions of the brain, but existing studies have paid insufficient attention to the link between the visual network and individual creativity. Given that visual networks are activated both when viewing physical objects and when imagining them, the ability to visually imagine may be an important component of creativity [43].

Comparing the EEG complexity of the metacontrol and creativity tasks, the EEG complexity of the creativity task was significantly higher than that of the metacontrol task at the same time scale (10–15) and remained slowly increasing at medium to high time scales, whereas the complexity of the creativity task remained stable or slightly decreased after reaching its peak. These results reveal differences in neural activity patterns between the two tasks: the metacontrol task relies primarily on neural network interactions across networks, whereas the creativity task relies more on communication between moderately distant sets of neurons within multiple functional brain networks. Both on moderate and high time scales, the creativity task involved more active communication between sets of neurons than the metacontrol task.

Notably, Takahashi reported that resting-state EEG complexity was significantly higher in patients with schizophrenia than in controls at higher time scales and that this complexity was reduced when patients were taking antipsychotic medications of the dopamine antagonist class [33]. Also, a significant association was found between grey matter volume of the dopamine system and individual creativity [30]. Furthermore, significant correlations between schizotypal personality and creativity have been found in non-affected relatives of schizophrenic patients and in the general population [44,45]. Clinical manifestations of the hyperactivity disorder (ADHD) subtype have been found to be associated with enhanced divergent thinking [46]. In psychiatric patients, increased EEG complexity is highly associated with cognitive decline [47]. Some researchers have suggested that cognitive decline in psychiatric disorders may result from a reduction in latent inhibition and that this ‘disinhibition’ provides a more flexible state, which in turn improves divergent thinking and creative performance [48]. This disinhibition is closely linked to the dopamine system, which is key to the regulation of different metacontrol states, and thus dopamine circuits may be one of the neurophysiological bases shared by metacontrol and creativity.

The limitations of this study are as follows: first, the sample source was relatively homogeneous, selected only from university students, and did not include other age groups. Second, due to the low spatial resolution of EEG technology, it was not possible to precisely locate the neural networks involved in creative activities. Future studies could consider using techniques such as functional magnetic resonance imaging (fMRI) for more in-depth analyses.

## 5. Conclusions

The introduction of multiscale entropy (MSE) analysis provides a new perspective for the study of the relationship between metacontrol and creativity. The findings reveal the moderating role of metacontrol ability in the process of creative thinking and the common basis of metacontrol and creative thinking at the neurophysiological level. Specifically, the high-metacontrol group performed significantly better than the low-metacontrol group in performing a creativity task. At higher time scales (10–16), the high-metacontrol group had significantly higher EEG complexity in bilateral temporal lobe regions than the low-metacontrol group. In addition, the high-creativity group had significantly higher EEG complexity in parietal and occipital regions than the low-creativity group. Both metacontrol task adaptation scores and creativity task scores were significantly and positively correlated with multi-electrode point EEG complexity. These findings emphasise the importance of metacontrol in facilitating creative thinking and point to the fact that both metacontrol and creativity are dependent on the activation of remote neural networks.

## 6. Limitation and Preference

Creative activities involve multiple psychological processes, including perception, memory, association, and reasoning [49,50]. This study analyzes creative thinking as a whole without further dividing it internally, so it is unclear whether the differences in EEG complexity are reflected in specific psychological activities or in every psychological activity. Future research can complement this. Furthermore, the limited spatial resolution of the EEG technique precluded precise localization of the neural networks engaged in the creative process. Further research could employ techniques such as functional magnetic resonance imaging (fMRI) to facilitate a more comprehensive analysis.

The complexity of EEG has been extensively employed in the investigation of mental disorders [33,47]. However, there is currently a paucity of studies utilising EEG complexity to examine cognitive processes. In this case, a comparison of brain activity between patients with developmental disorders (especially those with severe cognitive impairments) and healthy individuals will facilitate the acquisition of new insights into the neural mechanisms underlying these cognitive processes. For example, individuals with autism often display decreased creativity and more specialised brain connectivity patterns [51,52]. Patients with bipolar disorder demonstrate a notable enhancement in creativity during manic episodes [53]. By studying these issues, we can gain further insight into the neural mechanisms that underpin creativity.

## Figures and Tables

**Table 1:** Multiscale entropy comparison of brain networks for metacontrol tasks at time scales of 10–15. Values with significant differences are marked in bold.

Network	*t*	*p*	Cohen’s d
DMN	1.56	0.062	
DAN	**1.85**	**0.035**	0.363
FMN	1.31	0.097	
LN	1.39	0.085	
SMN	1.31	0.098	
VAN	1.14	0.13	
VN	1.05	0.149	

**Table 2:** Comparison of multiscale entropy of AUT brain networks at the time scale of 10–15. Values with significant differences are marked in bold.

Network	*t*	*p*	Cohen’s d
DMN	**3.19**	**0.001**	0.626
DAN	**3.2**	**0.001**	0.628
FMN	**2**	**0.025**	0.392
LN	**2.28**	**0.013**	0.447
SMN	**3.08**	**0.002**	0.604
VAN	**2.67**	**0.005**	0.524
VN	**3.36**	**<0.001**	0.659

## Data Availability

The data presented in this study are available on request from the corresponding author due to the privacy of the subjects.

## References

[1] Beaty R.E., Benedek M., Barry K.S., Silvia P.J. (2015). Default and executive network coupling supports creative idea production. Sci. Rep..

[2] Goschke T., Bolte A. (2014). Emotional modulation of control dilemmas: The role of positive affect, reward, and dopamine in cognitive stability and flexibility. Neuropsychologia.

[3] Del Giudice M., Crespi B.J. (2018). Basic functional trade-offs in cognition: An integrative framework. Cognition.

[4] Musslick S., Cohen J.D. (2020). Rationalizing constraints on the capacity for cognitive control. Trends Cogn. Sci..

[5] Akbari C.S., Hommel B. (2012). Creative mood swings: Divergent and convergent thinking affect mood in opposite ways. Psychol. Res..

[6] Carsten K.W., Dreu D., Bernard A., Nijstad M.B., Inge W., Marieke R. (2012). Working Memory Benefits Creative Insight, Musical Improvisation, and Original Ideation Through Maintained Task-Focused Attention. Personal. Soc. Psychol. Bull..

[7] Hommel B. (2015). Between persistence and flexibility: The Yin and Yang of action control. Adv. Motiv. Sci..

[8] Hsieh Y., Kirschner K., Copland M. (2021). Improving outcomes in chronic myeloid leukemia through harnessing the immunological landscape. Leukemia.

[9] Eppinger B., Goschke T., Musslick S. (2021). Meta-control: From psychology to computational neuroscience. Cogn. Affect. Behav. Neurosci..

[10] Arden R., Chavez R.S., Grazioplene R., Jung R.E. (2010). Neuroimaging creativity: A psychometric view. Behav. Brain Res..

[11] Dietrich A., Kanso R. (2010). A review of EEG ERP and neuroimaging studies of creativity and insight. Psychol. Bull..

[12] Jung R.E., Mead B.S., Carrasco J., Flores R.A. (2013). The structure of creative cognition in the human brain. Front. Hum. Neurosci..

[13] Tononi G., Edelman G.M., Sporns O. (1998). Complexity and coherency: Integrating information in the brain. Trends Cogn. Sci..

[14] Abarbanel H.D., Rabinovich M.I. (2001). Neurodynamics: Nonlinear dynamics and neurobiology. Curr. Opin. Neurobiol..

[15] Vakorin V.A., Lippe S., McIntosh A.R. (2011). Variability of brain signals processed locally transforms into higher connectivity with brain development. J. Neurosci..

[16] McDonough I.M., Nashiro K. (2014). Network complexity as a measure of information processing across resting-state networks: Evidence from the Human Connectome Project. Front. Hum. Neurosci..

[17] Garrett D.D., Samanez-Larkin G.R., MacDonald S.W.S., Lindenberger U., McIntosh A.R., Grady C.L. (2013). Moment-to-moment brain signal variability:a next frontier in human brain mapping?. Neurosci. Biobehav. Rev..

[18] Ghanbari Y., Bloy L., Edgar J.C., Blaskey L., Verma R., Roberts T.P.L. (2015). Joint analysis of band-specific functional connectivity and signal complexity in autism. J. Autism Dev. Disord..

[19] Faisal A.A., Selen L.P.J., Wolpert D.M. (2008). Noise in the nervous system. Nat. Rev. Neurosci..

[20] Richman J.S., Lake D.E., Moorman J.R., Michael L.J., Ludwig B. (2004). Sample entropy. Methods Enzymol..

[21] Takahashi T. (2013). Complexity of spontaneous brain activity in mental disorders. Prog. Neuropsychopharmacol. Biol. Psychiatry.

[22] Bocanegra B.B., Hommel B. (2014). When Cognitive Control Is Not Adaptive. Psychol. Sci..

[23] Von S.A., Sarnthein J. (2000). Different frequencies for different scales of cortical integration: From local gamma to long range alpha/theta synchronization. Int. J. Psychophysiol..

[24] Kowatari Y., Lee S.H., Yamamura H., Nagamori Y., Levy P., Yamane S. (2009). Neural networks involved in artistic creativity. Hum. Brain Mapp..

[25] Costa M., Goldberger A.L., Peng C.K. (2005). Multiscale entropy analysis of biological signals. Phys. Rev. E Stat. Nonlinear Soft Matter Physic..

[26] Takeuchi H., Taki Y., Sassa Y., Hashizume H., Sekiguchi A., Fukushima A. (2010). Regional gray matter volume of dopaminergic system associate with creativity: Evidence from voxel-based morphometry. Neuroimage.

[27] Clayson P.E., Carbine K.A., Baldwin S.A., Larson M.L. (2019). Methodological reporting behavior, sample sizes, and statistical power in studies of event-related potentials: Barriers to reproducibility and replicability. Psychophysiology.

[28] Zink N., Bensmann W., Arning L., Colzato L.S., Stock A.K., Beste C. (2019). The role of DRD1 and DRD2 receptors for response selection under varying complexity levels: Implications for metacontrol processes. Int. J. Neuropsychopharmacol..

[29] Zink N., Stock A.K., Vahid A., Beste C. (2018). On the neurophysiological mechanisms underlying the adaptability to varying cognitive control demands. Front. Hum. Neurosci..

[30] Runco M.A., Acar S. (2012). Divergent thinking as an indicator of creative potential. Creat. Res. J..

[31] Rominger C., Papousek I., Perchtold C.M., Benedek M., Weiss E.M., Schwerdtfeger A., Fink A. (2019). Creativity is associated with a characteristic U-shaped function of alpha power changes accompanied by an early increase in functional coupling. Cogn. Affect. Behav. Neurosci..

[32] Richman J.S., Moorman J.R. (2000). Physiological time-series analysis using approximate entropy and sample entropy. Am. J. Physiol.-Heart Circ. Physiol..

[33] Takahashi T., Raymond Y.C., Mizunoa T., Kikuchic M., Murata T., Takahashi K., Wada Y. (2010). Antipsychotics reverse abnormal EEG complexity in drug-naive schizophrenia: A multiscale entropy analysis. NeuroImage.

[34] Ueno K., Takahashi T., Takahashi K., Mizukami K., Tanaka Y., Wada Y. (2015). Neurophysiological basis of creativity in healthy elderly people: A multiscale entropy approach. Clin. Neurophysiol..

[35] Takahashia T., Raymond Y.C., Murataa T., Mizuno T., Kikuchi M., Mizukami K., Kosaka H., Takahashi K., Wada Y. (2009). Age-related variation in EEG complexity to photic stimulation: A multiscale entropy analysis. Clin. Neurophysiol..

[36] Lewandowska M., Tołpa K., Rogala J., Piotrowski T., Dreszer J. (2023). Multivariate multiscale entropy (mMSE) as a tool for understanding the resting-state EEG signal dynamics: The spatial distribution and sex/gender-related differences. Behav. Brain Funct..

[37] Giacometti P., Perdue K.L., Diamond S.G. (2014). Algorithm to find high density EEG scalp coordinates and analysis of their correspondence to structural and functional regions of the brain. J. Neurosci. Methods.

[38] Jung R.E., Grazioplene R., Caprihan A., Chavez R.S., Haier R.J. (2010). White matter integrity creativity and psychopathology: Disentangling constructs with diffusion tensor imaging. Public Libr. Sci. ONE.

[39] Levine M.S., Chen J.Y., Wang E.A., Cepeda C. (2013). Dopamine imbalance in Huntington’s disease: A mechanism for the lack of behavioral flexibility. Front. Neurosci..

[40] Takeuchi H., Taki Y., Hashizume H., Sassa Y., Nagase T., Nouchi R. (2012). The association between resting functional connectivity and creativity. Cereb. Cortex.

[41] Beaty R.E., Seli P., Schacter D.L. (2019). Network neuroscience of creative cognition: Mapping cognitive mechanisms and individual differences in the creative brain. Curr. Opin. Behav. Sci..

[42] Schnitzler A., Gross J. (2005). Normal and pathological oscillatory communication in the brain. Nat. Rev. Neurosci..

[43] Breedlove J.L., St-Yves G., Olman C.A., Naselaris T. (2020). Generative Feedback Explains Distinct Brain Activity Codes for Seen and Mental Images. Curr. Biol..

[44] Andreasen N.C. (1987). Creativity and mental illness: Prevalence rates in writers and their first-degree relatives. Am. J. Psychiatry.

[45] Kyaga S., Landen M., Boman M., Hultman C.M., Langstrom N., Lichtenstein P. (2013). Mental illness suicide and creativity: 40-year prospective total population study. J. Psychiatr. Res..

[46] Boot N., Baas M., van Gaal S., Cools R., De Dreu C.K. (2017). Creative cognition and dopaminergic modulation of fronto-striatal networks: Integrative review and research agenda. Neurosci. Biobehav. Rev..

[47] Mizuno T., Takahashi T., Cho R.Y., Kikuchi M., Murata T., Takahashi K. (2010). Assessment of EEG dynamical complexity in Alzheimer’s disease using multiscale entropy. Clin. Neurophysiol..

[48] Carson S.H., Peterson J.B., Higgins D.M. (2003). Decreased latent inhibition is associated with increased creative achievement in high-functioning individuals. J. Personal. Soc. Psychol..

[49] Sternberg R.J. (1999). Handbook of Creativity.

[50] Runco M.A., Jaeger G.J. (2012). The standard definition of creativity. Creat. Res. J..

[51] Happé F.G.E. (1993). Communicative competence and theory of mind in autism: A test of relevance theory. Cognitive.

[52] Ursino M., Serra M., Tarasi L., Ricci G., Magosso E., Romei V. (2022). Bottom-up vs. top-down connectivity imbalance in individuals with high-autistic traits: An electroencephalographic study. Front. Syst. Neurosci..

[53] Santosa C.M., Strong C.M., Nowakowska C., Wang P.W. (2007). Enhanced creativity in bipolar disorder patients: A controlled study. J. Affect. Disord..

